# Emergency response in water, sanitation and hygiene to control cholera in post-earthquake Nepal in 2016

**DOI:** 10.2166/washdev.2018.016

**Published:** 2018-07-16

**Authors:** Kazutaka Sekine, Mellisa Roskosky

**Affiliations:** 1 UNICEF Sierra Leone, Freetown, Sierra Leone; 2 Johns Hopkins University, Baltimore, Maryland, USA

**Keywords:** cholera, diarrhea, disasters, disease outbreaks, sanitation

## Abstract

After the 2015 earthquake in Nepal that killed approximately 9,000 people, the country faced an increased risk of cholera outbreaks due to extensive destruction of water and sanitation infrastructure and massive displacement. The disaster revealed long-standing weaknesses in water and sanitation systems in the country. Anticipating a cholera outbreak in 2016, UNICEF, Johns Hopkins University, and the Group for Technical Assistance partnered to support the Government of Nepal to ensure a safe water supply and improve sanitation and hygiene. This article discusses challenges, gaps, lessons learned and recommendations that were drawn from the authors' experience in cholera prevention and control in post-earthquake Nepal. Challenges identified include lack of regular water quality testing and monitoring, inconsistent use of point-of-use water treatment products, and lack of a fast-track mechanism for rapid response. The article argues for building a resilient water and sanitation system to secure sustainable and equitable access to safe drinking water.

## PROBLEM

On 25 April 2015, an earthquake of magnitude 7.8 hit central Nepal. The effect of the earthquake was catastrophic, killing nearly 9,000 and injuring more than 22,000 people. Over 5 million people living in 31 districts were affected (National Planning Commission 2015). It also inflicted grave damage on 7,741 water supply schemes and approximately 388,000 sanitation facilities, leading to deterioration of access to safe drinking water and proper sanitation in earthquake-affected areas (National Planning Commission 2015). Displacement was fueled by a powerful aftershock of magnitude 7.3 on 12 May 2015 and the danger of collapsing damaged houses. Over 100 displacement sites hosted affected people in need of a temporary shelter (Nepal Earthquake Assessment Unit 2015). With nearly 600,000 homes destroyed and another 280,000 damaged by the quakes (National Planning Commission 2015), hundreds of thousands of affected people were forced to stay in makeshift shelters (OCHA 2015) where access to clean water was limited, and toilets were overwhelmed. These circumstances in the post-earthquake period enhanced the likelihood of outbreaks of waterborne diseases, including cholera.

## LOCAL SETTING

Cholera, an infectious disease caused by *Vibrio* *cholerae* *,* is endemic in Nepal. Outbreaks often occur during the annual monsoon season (Ise *et al*. 1996; Gautam *et al*. 2010; Karki *et al*. 2010; Bhandari & Bhusal 2013; Dixit *et al*. 2014; Pandey 2015; Thapa Shrestha *et al*. 2015). In 2016 in the aftermath of the natural disaster, the country experienced a cholera outbreak with a total of 169 confirmed cases. Of those cases, 150 were detected within the Kathmandu valley. The transmission of *V. cholerae* and other waterborne diseases in Nepal is associated with a poor water supply system. The bacterium is known to be present in both drinking water and surface water in the valley and other regions in the country (Dixit *et al*. 2014), which was confirmed through environmental surveillance in 2016. The water supply infrastructure of Kathmandu is old with many breakages (Sharma 2006; Dixit *et al*. 2014). The maintenance and expansion of water and sanitation infrastructure have not kept pace with the rapidly growing urban population (Muzzini & Aparicio 2013). Various water sources are contaminated with fecal bacteria due to a combination of the leaking water sewage system and the pervasive practice of open defecation in impoverished communities. In Nepal, 71% of people use unsuitable sources of drinking water which are contaminated with *E. coli* (>1 cfu/100 mL) (Central Bureau of Statistics 2015). Furthermore, due to the unreliability of municipal pipelines, people living in the Kathmandu valley rely on a range of other options, including tap water, water jars, private water tanks, traditional stone spouts (an ancient water distribution network using gravity and rainwater), and dug wells.

## APPROACH

The Government of Nepal, in collaboration with UNICEF, Johns Hopkins University (Baltimore, MD, USA), and the Group for Technical Assistance (Kathmandu, Nepal), implemented a multi-sectoral response to contain the cholera outbreak in 2016. In this article, we present viewpoints and insights into cholera prevention and control based on our experience during that outbreak. From a pragmatic perspective, the article outlines challenges, gaps, lessons learned and recommendations that were drawn from our cholera response to ensure a safe water supply and to improve sanitation and hygiene.

During the outbreak response, the three organizations above launched awareness-raising campaigns through community engagement in transmission hotspots where a cluster of cholera cases was detected. The campaigns targeted households, food outlets (e.g. restaurants, tea shops, street-food vendors), and schools. Door-to-door visits were carried out by Female Community Health Volunteers to enhance risk communication and hygiene promotion while distributing and demonstrating proper use of point-of-use (POU) water treatment products and soap. In addition, prevention messages were aired on the radio and were spread through vehicles equipped with amplification equipment. These information and awareness campaigns focused on five key messages: household water treatment, safe storage of water at home, handwashing, food hygiene, and basic sanitation.

In addition, water quality testing (e.g. chlorine residual test and presence-absence fecal coliform test) was performed in areas perceived as hotspots, and the results of the tests were mapped out using the geographic information system to characterize the distribution of contaminated water that we identified. Sharing of the results of that testing with communities was carried out by government water suppliers, youth volunteers with non-governmental organizations (NGOs), Female Community Health Volunteers, and public volunteers deployed in municipalities. The results of simplified presence-absence fecal coliform tests were shared to sensitize community members on fecal contamination of their drinking water in the hope that this would trigger behavioral changes towards water treatment. Our monitoring activities suggested that communities accepted the results as easy to understand and compelling evidence of fecal contamination. In addition, government water suppliers and water tanker associations were trained on water chlorination and testing methods.

## CHALLENGES AND GAPS

Chlorination of water supplied by the government was closely monitored during the outbreak. Outside of tap water, regular water quality testing and monitoring were non-existent, and this posed a major challenge in the Kathmandu valley, where a number of water suppliers, such as private water tankers and community-based water suppliers, operate. This gap made the mapping of water suppliers, and the implementation of regulatory and supervisory activities, very difficult. Lack of government guidelines on water chlorination was another major issue hampering the response.

While POU chlorination of drinking water is an effective, low-cost, easy-to-use method for disinfecting contaminated drinking water (Clasen *et al.* 2007), the successful application and continued use of POU chlorine products hinges on the end user’s behavior. Our monitoring of household water treatment practices and the results of residual chlorine testing indicated that the proper use of POU chlorine products, and safe storage of drinking water within the household, remained low following the campaigns due to lack of knowledge about the proper use of POU chlorine products and the risk of diarrheal diseases perceived as being low. This may suggest the need for innovative water and sanitation interventions, including widespread social marketing of water purification and safe storage products to ensure the continued practice of the promoted behaviors.

Lack of a fast-track funding mechanism in the local government to mobilize a quick response led to some delays in implementing planned interventions. Further, weak coordination and suboptimal information sharing across the Health and WASH sectors was recognized as a challenge. Convoluted division of responsibilities between the Health and WASH sectors also created some confusion. For example, although the implementation of water chlorination was led by district health authorities, it was unclear which government agency had the authority for it.

## RECOMMENDATIONS

Establishing a robust system for water quality testing and monitoring and developing national guidelines on water chlorination should be acknowledged as a priority for cholera prevention. Since making a lasting change in preventive behaviors for cholera usually involves substantial time, the communication component should be mainstreamed as part of regular WASH programs. Strengthening sector-wide approaches and a coordination mechanism will require greater attention and effort from all partners. Clarification needs to be made regarding the responsibilities of government agencies in the Health and WASH sectors in the implementation of a response to cholera outbreaks.

Stronger political and financial commitments at a national level are required for continued implementation of a multi-sectoral strategy for cholera prevention and control. A government contingency budget for rapid response in the case of an outbreak needs to be sought. Moreover, enhanced engagement of district and municipality governments in epidemic preparedness is essential for successful planning and management of cholera interventions at district and community levels.

## CONCLUSION

Without a control group, it is difficult to establish whether the cholera prevention and control measures, which were undertaken in post-earthquake Nepal, were the driving force behind the outbreak subsiding. However, no cholera cases were reported from the temporary displacement sites, and no deaths due to cholera were reported from sentinel site hospitals in 2016. It is also important to note that prevention of cholera recurrence was witnessed after implementation of the targeted interventions and that there was no major response implemented except the one discussed in the article. The partnership that enabled the rapid implementation of a contingency plan was key to controlling the outbreak in the post-disaster context. Yet, government-led coordination across the Health and WASH sectors for cholera response needs to be strengthened to increase efficiency and timeliness of response activities at different levels.
